# Bioinformatics and Functional Validation of *CqPRX9L1* in *Chenopodium quinoa*

**DOI:** 10.3390/plants14142246

**Published:** 2025-07-21

**Authors:** Hongxia Guo, Linzhuan Song, Yufa Wang, Li Zhao, Chuangyun Wang

**Affiliations:** College of Agriculture, Shanxi Agricultural University, Taiyuan 030031, China; hongxiaguo@sxau.edu.cn (H.G.); 19833909366@163.com (L.S.); 19149350418@163.com (Y.W.); lizhao@sxau.edu.cn (L.Z.)

**Keywords:** *Chenopodium quinoa*, *CqPRX9L1*, drought stress, heterologous expression

## Abstract

As a plant-specific peroxidase family, class III peroxidase (PRX) plays an important role in plant growth, development, and stress response. In this study, a preliminary functional analysis of *CqPRX9L1* was conducted. Bioinformatics analysis revealed that *CqPRX9L1* encodes a 349-amino acid protein belonging to the plant-peroxidase-like superfamily, featuring a transmembrane domain and cytoplasmic localization. The promoter region of *CqPRX9L1* harbors various cis-acting elements associated with stress responses, hormone signaling, light regulation, and meristem-specific expression. The tissue-specific expression pattern of the *CqPRX9L1* gene and its characteristics in response to different stresses were explored using subcellular localization, quantitative real-time PCR (qRT-PCR), and heterologous transformation into *Arabidopsis thaliana.* The results showed that CqPRX9L1, with a transmembrane structure, was localized in the cytoplasm, which encodes 349 amino acids and belongs to the plant-peroxisome-like superfamily. The promoter region contains stress-response elements, hormone-response elements, light-response elements, and meristem expression-related elements. The expression of *CqPRX9L1* was relatively higher in ears and roots at the panicle stage than in stems and leaves. *CqPRX9L1* showed a dynamic expression pattern of first decreasing and then increasing under abiotic stresses such as 15% PEG 6000, low temperature, and salt damage, with differences in response time and degree. *CqPRX9L1* plays an important role in response to abiotic stress by affecting the activity of antioxidant enzymes such as superoxide dismutase (SOD) and peroxidase (POD), as well as the synthesis and decomposition of proline (Pro). *CqPRX9L1* also affects plant bolting and flowering by regulating key flowering genes (such as FT and AP1) and gibberellin (GA)-related pathways. The results establish a foundation for revealing the functions and molecular mechanisms of the *CqPRX9L1* gene.

## 1. Introduction

Peroxidases (POD; EC 1.11.1. X) are an important enzyme family that can catalyze various substrate oxidation reactions. These enzymes have been widely found in animals, plants, and microorganisms [1]. Based on protein structures and catalytic properties, peroxidases are classified into three categories: class I (ascorbic acid peroxidase), class II (lignin peroxidase), and class III (secretory peroxidase) (PRX EC 1.11.1.7) [2,3]. PRXs are plant-specific secreted glycoproteins [4,5,6]. As of now, up to 8998 PRX have been included in the RedoxiBase database, accounting for approximately 75% of the total non-animal PODs [7]. It is known that there are 138 and 73 PRX members in rice and *Arabidopsis*, respectively, but most of their functions are still unclear [8,9,10]. PRXs are mainly localized in extracellular spaces [11] or vacuoles, and some PRX analogs are predicted to be localized in mitochondria or bound to the cell membrane [12]. PRX family members, which contain disulfide bonds, calcium ions, and N-terminal signal peptides [13], are heme-dependent and capable of undergoing glycosylation. PRXs play a crucial role in the plant life cycle and participate in the regulation of plant physiological activities extensively. PRXs have many isoenzymes with different catalytic characteristics, which can not only participate in plant hormone metabolism [14], seed germination, cell wall protein cross-linking, lignin synthesis [15], maintaining cell growth and elongation [12,16,17], fruit development, ripening, senescence, and other plant growth and development processes [18], but also play an important role in responding to biotic and abiotic stresses [3,17,19,20]. PRX can oxidatively polymerize lignin monomers and other components of plant cell walls, such as suberin and extensin. *PRX9* and *PRX40* maintain the integrity of the tapetum and microspore cell wall during another development in *Arabidopsis thaliana* [11]. The loss of *PRX30* function enhances rice resistance to bacterial infection [21]. *GhPOX* is significantly up-regulated during cotton fiber elongation [22]. Studies on foxtail millet [4], Arabidopsis thaliana [6], maize [23], potato [24], and rice [9,10,19] have found that most PRX genes with drought resistance functions have higher transcription levels in plant roots. For example, in foxtail millet, *SitPRX12*, *SitPRX33*, *SitPRX49*, *SitPRX110*, and *SitPRX126* are highly expressed in roots and significantly increased under PEG stress, indicating that the *SitPRX* gene family may be closely related to plant root development and drought stress response. The expressions of *ZmPRX26* and *ZmPRX75* are relatively higher in roots and significantly up-regulated under drought stress and PEG stress. In *Arabidopsis thaliana*, *AtPrx62* and *AtPrx69* can promote root hair growth under low-temperature conditions [25], while *AtPRX3* (ATRCI3) is positively regulated under drought and salt stress [26]. In *Brachypodium distachyon*, *BdPrx085* gene expression is down-regulated under NaCl and PEG treatment, whereas *BdPrx064* exhibits higher expression levels under PEG treatment [27]. The overexpression of *AtPrx22*, *AtPrx39*, and *AtPrx69* increased cold tolerance in the BRI1-GFP plants [28].

Under arsenic (As) stress conditions, the overexpression of rice *OsPRX38* in Arabidopsis increased PRX, SOD (superoxide dismutase), and GST (glutathione S-transferase) activities and enhanced lignification, leading to a decrease in As accumulation [29]. Under drought stress, the expressions of *TaPrx01*, *TaPrx03*, *TaPrx19*, *TaPrx68*, *TaPrx107*, and *TaPrx109-C* decreased [30]. *BrPRX* plays an important role in light response, hormone regulation, stress resistance, and growth and development [31]. 

*Chenopodium quinoa* is an annual dicotyledonous crop that originated in the high-altitude region of the Andes Mountains in South America. It is rich in eight essential amino acids for humans and one essential amino acid for infants and young children [19]. Notably, the Food and Agriculture Organization of the United Nations (FAO) has identified quinoa as the only plant-based “complete nutritional food” capable of meeting fundamental human dietary requirements [32]. Meanwhile, quinoa also has outstanding resistance to abiotic stresses such as drought, low temperature, and salt injury. Due to its outstanding nutritional value and resistance to abiotic stress, quinoa has attracted widespread attention. Especially in Shanxi province, where there is limited rainfall and water scarcity, drought has become one of the most important abiotic factors restricting agricultural development [33]. Therefore, it is particularly important to explore genes related to drought resistance in quinoa and analyze their drought resistance mechanisms.

In our previous transcriptomic studies on wild quinoa, we analyzed differentially expressed genes (DEGs) under varying concentrations of PEG 6000-induced drought stress (5%, 10%, 15%, and 20%). As the PEG concentration increased from 5% to 20%, the number of down-regulated genes rose sharply from 583 to 5678 (Table 1).

To systematically analyze the key metabolic pathways involved in quinoa’s drought response, we performed KEGG enrichment analysis on the top 10 significantly enriched pathways (ranked by *p*-value) from each of the four PEG 6000 treatment groups. This comparative approach revealed both conserved and concentration-specific pathway activation patterns:

Further pathway enrichment analysis demonstrated that the differentially expressed genes (DEGs) from all PEG 6000 treatment groups showed significant enrichment in the phenylpropanoid biosynthesis pathway (ath00940), which represents a crucial metabolic route involved in plant stress adaptation and defense mechanisms. suggesting its pivotal role in quinoa’s response to drought stress. Strikingly, *CqPRX9L1* was one of only three genes (belonging to the peroxidase family) that displayed significant differential expression in all four treatment groups.

The 15% PEG 6000 treatment was identified as the critical concentration, inducing 1971 up-regulated and 3382 down-regulated genes—a substantial increase compared to lower concentrations (5% and 10%). At this level, the number of down-regulated genes surpassed up-regulated genes for the first time. Additionally, 15% PEG 6000 elicited stronger enrichment in stress-related pathways (phenylpropanoid biosynthesis) than higher concentrations (20%), while avoiding severe metabolic disruption. This makes it an optimal condition for identifying core drought-responsive regulators.

Notably, a drought-responsive gene, designated as *CqPRX9L1* (LOC_110724764), is down-regulated under stress conditions and belongs to the plant-peroxidase-like superfamily. PRXs play an important role in plant growth, development, and stress response; however, expression patterns and functions of PRXs in quinoa have rarely been reported, and their biological functions remain unclear. In this study, bioinformatics techniques were utilized to analyze the structure, promoter elements, phylogenetic relationships, and physicochemical properties of this gene. Additionally, qRT-PCR technology is used to analyze the expression patterns of *CqPRX9L1* in different tissues and different stress treatments. Combined with physiological indexes related to drought resistance and phenotypes, the expression characteristics and functions were preliminarily studied, providing a theoretical basis for further revealing the biological functions of *CqPRX9L1*.

## 2. Results and Discussion

### 2.1. Bioinformatic Analysis of CqPRX9L1

**Figure 1:**
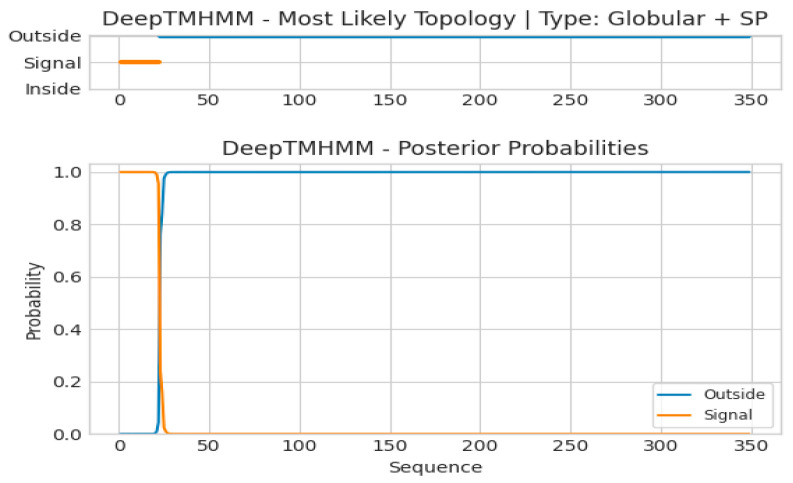
Transmembrane structure analysis.

**Figure 2:**
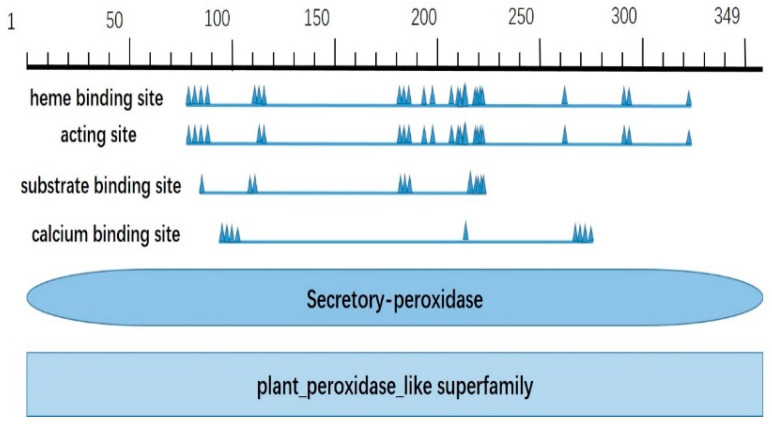
Conservative structural domain analysis. The scale bar (shown in the upper part) represents the length of the CqPRX9L1 amino acid.

**Figure 3:**
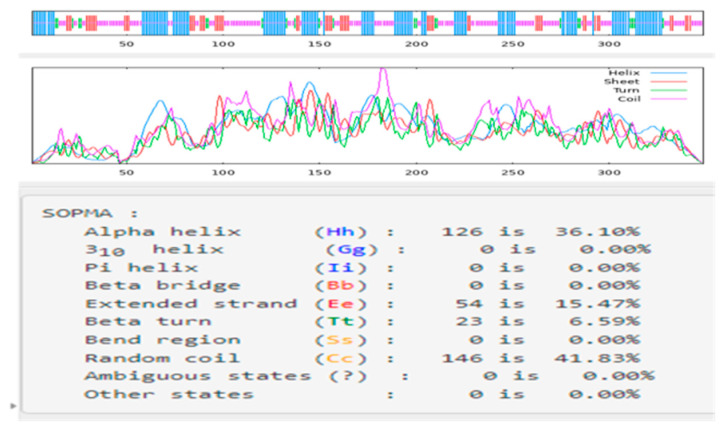
Secondary structure of protein. Different colors represent different secondary structures and “?”represents the region where structures such as α-helix and extended chain cannot be unambiguously determined.

**Figure 4:**
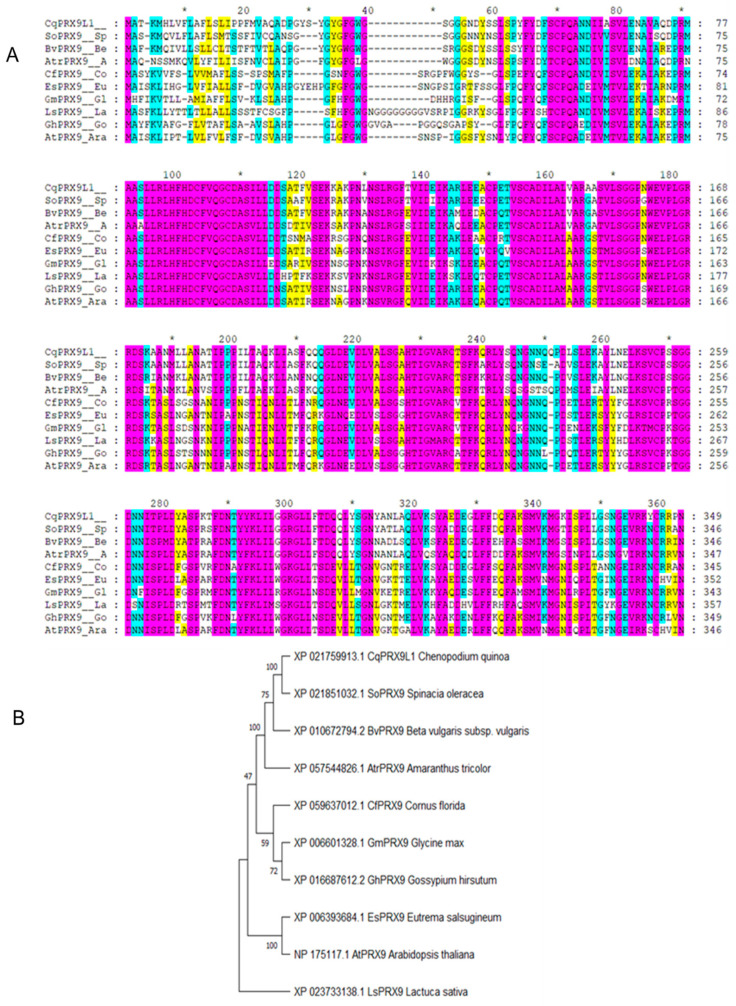
Amino acid sequence alignment and evolutionary relationship of CqPRX9L1 and its homologous proteins. (**A**) Amino-acid sequence alignment of CqPRX9L1 and its homologsProteins. The red, blue and yellow backgrounds represent amino acid similarities of 100%, over 80% and over 60%, respectively. (**B**) evolutionary relationship analysis of CqPRX9L1. * represent a marker line, serving as a spacing marker for sequence line numbers.

*CqPRX9L1* encodes 349 amino acids, with a relative molecular weight of 37.95 kD and a theoretical isoelectric point of 5.86, indicating that it is an acidic protein; the theoretical derivation shows that the half-life period is greater than 10 h, and the unstable parameter is 45.6, which belongs to the unstable protein category; the amino acids with higher relative content are Leu (41, 11.7%), Ala (35, 10%), Ser (31, 8.9%), Gly (27, 7.7%), Asn (20, 5.7%), Asp (20, 5.7%), Pro (19, 5.4%), Val (18, 5.2%), Phe (17, 4.9%), and Gln (16, 4.6%); the total negatively charged residue (Asp + Glu) is 33; the total positively charged residue (Arg + Lys) is 30, and the average hydrophilicity of the protein is −0.105, indicating it is a hydrophilic protein; and the transmembrane analysis showed that the protein has one transmembrane structure (Figure 1). The results of conservative domain analysis indicate that Cq*PRX9L1* belongs to the plant peroxidase superfamily (Figure 2); the secondary structural analysis of protein showed that the random coil and α-helix accounted for 41.83% and 36.1%, respectively, while the proportion of extended chains and β-fold were 15.47% and 6.59%, respectively (Figure 3); and the subcellular localization prediction suggests that Cq*PRX9L1* may be localized in the cytoplasm. The analysis of the 2.5 kb sequence upstream of the promoter indicates that the cis-acting elements in the promoter region of the *CqPRX9L1* gene can be classified into the following categories according to their functional annotations: 1. light-responsive elements, such as the TCT motif, ATCT motif, MRE, etc.; 2. stress-responsive elements, such as MBS, LTR, DRE1, and the W box; 3. hormone-responsive elements, such as the TCA element, ABRE, TGACG motif, etc.; and 4. The meristem-specific element CAT box, indicating that the *CqPRX9L1* gene may be regulated by light and plant hormones and is related to tissue-specific expression and stress response (Table 2). The amino acid sequence alignment and phylogenetic tree analysis of *CqPRX9L*1 indicate that *CqPRX9L1* is closely related to spinach (*Spinacia oleracea*) (So PRX9, XP_021851032.1) and sugar beet (*Beta vulgaris*) (Bv PRX9, XP_010672794.2) (Figure 4).

### 2.2. Expression Analysis of CqPRX9L1 in Quinoa

#### 2.2.1. Spatiotemporal Expression Pattern Analysis of CqPRX9L1

**Figure 5:**
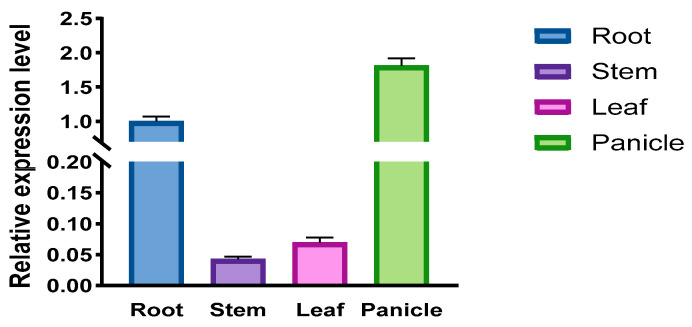
Expression pattern analysis of *CqPRX9L1* in different tissues under normal. Expression pattern of *CqPRX9L1* in different tissues under normal growth conditions. *Actin1* was selected as the reference gene. Three biological replicates were used.

To characterize the expression profiling of *CqPRX9L1*, we performed quantitative real-time PCR (qRT-PCR) analysis using RNA isolated from distinct quinoa tissues (roots, stems, mature leaves, and developing spikes [1.3–1.8 cm in length]) collected at the spike emergence stage. The results revealed pronounced tissue-specific expression of CqPRX9L1 (Figure 5), displaying significantly elevated transcript accumulation in spikes (25.7-fold) and root tissues (14.3-fold) relative to leaves. This differential expression pattern implies potential involvement in inflorescence morphogenesis and root-specific physiological processes. These results are consistent with the findings of Zhai et al. (2024) that *ZmPRX1* expression was higher in roots than in other tissues [34].

#### 2.2.2. Expression Analysis of CqPRX9L1 Under Different Stress Treatments

**Figure 6:**
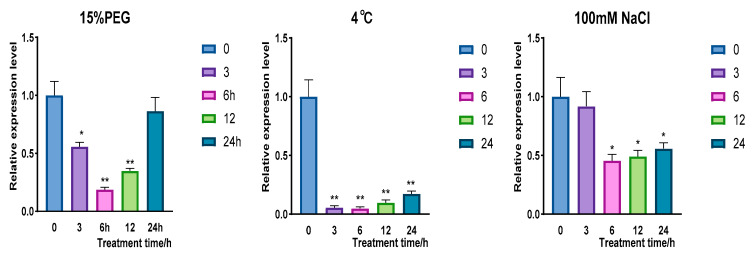
Analysis of *CqPRX9L1* in response to different stress treatments. Actin1 was selected as the reference gene. *n* = 3. The asterisk (*) symbols indicate significant differences relative to 0 h. * *p* < 0.05, ** *p* < 0.01.

To collect leaves treated with 15% PEG 6000 at 0 h, 3 h, 6 h, 12 h, and 24 h and analyze the expression pattern of *CqPRX9L1* utilizing qRT-PCR technologies. The results showed that the expression of *CqPRX9L1* first decreases and then increases under 15% PEG 6000 treatment. At the beginning of stress, the expression of *CqPRX9L1* is significantly down-regulated, reaching a minimum at 6 h, which is about 55% of that before treatment; the expression returns to 86% of that before treatment. The expression of *CqPRX9L1* is also significantly down-regulated under low-temperature treatment at 4 °C; the expression of *CqPRX9L1* is about 6% of that before treatment at 3 h; the expression of CqPRX9L1 also shows a trend of first decreasing and then increasing, reaching a minimum at 6 h, which is about 45% of that before treatment; and the expression of *CqPRX9L1* returns to about 56% of that before treatment (Figure 6). These results indicate that *CqPRX9L1* regulates functional genes negatively downstream under various abiotic stresses such as 15% PEG 6000 treatment, low-temperature treatment, and salt injury, while the response time and degree were different. These results are consistent with previous findings demonstrating that *ZmPRX26*, *ZmPRX42*, *ZmPRX71*, and *ZmPRX75* were down-regulated by NaCl and PEG stress treatment [23].

**Figure 7:**
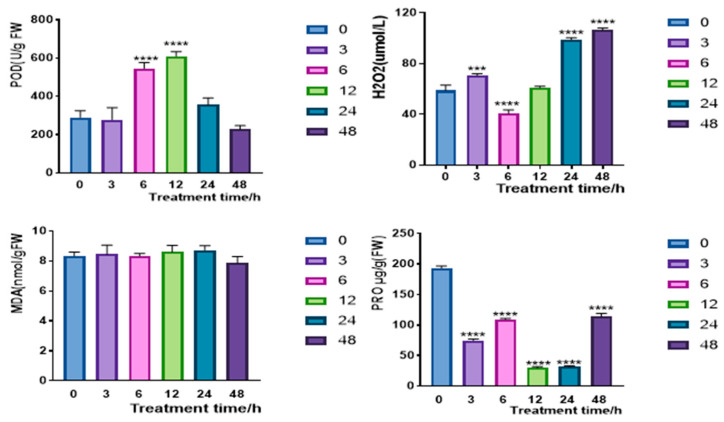
Effects of 15% PEG 6000 treatment on physiological indicators in quinoa plants. Actin1 was selected as the reference gene. *n* = 3. The asterisk (*) symbols indicate significant differences relative to 0 h. *** *p* < 0.001, **** *p* < 0.0001.

In order to analyze the intrinsic relationship between the expression pattern of *CqPRX9L1* and physiological response under abiotic stress, leaves were simultaneously taken at 0 h, 3 h, 6 h, 12 h, 24 h, and 48 h of 15% PEG 6000 treatment to detect changes in drought-related physiological indicators such as POD, SOD, MDA, and Pro. POD activity slightly decreased to 95.7% and reached a peak at 12 h, approximately 2.1-fold, and then decreased to 78% at 48 h. The H_2_O_2_ content was up-regulated at 3 h and reached 1.2-fold, then decreased to 69% at 6 h, and finally increased to 1.8-fold at 48 h, exhibiting a negative correlation with a temporal lag. POD activity showed a transient down-regulation, slightly deviating from the overall trend. These results indicate that *CqPRX9L1* may negatively regulate POD activity, for example, by regulating the transcription of POD genes [35] or regulating the stability of POD proteins to affect the antioxidant defense ability of plants under PEG stress. The transient down-regulation of POD activity at 3 h may be due to the preferential activation of faster defense mechanisms in the early stages of stress or the maintenance of an appropriate concentration of ROS as a signaling molecule to promote the establishment of plant stress defense mechanisms [36]. However, up-regulation at 6 h indicates that the plant has activated its defense mechanism by enhancing POD activity to decompose H_2_O_2_ and maintain ROS homeostasis. After 12 h, it may be down-regulated due to insufficient energy supply and decreased enzyme activity under long-term stress, leading to an increase in H_2_O_2_ accumulation. As a key indicator reflecting the degree of membrane lipid peroxidation, MDA showed no significant difference in its content before and after stress treatment, indicating that no significant peroxidation damage occurred to the membrane lipids under the current treatment concentration and time conditions. This suggests that the structure and function of the cell membrane remain relatively stable during stress, without severe damage to the membrane system caused by oxidative stress. The Pro accumulation decreased to 39% at 3 h and then returned to 57% at 6 h. At 12 h, the Pro content decreased to 15%, then returned to 59% at 48 h (Figure 7), showing a fluctuating pattern, indicating *CqPRX9L1* may have an impact on Pro content, but there is no significant correlation between the two changes, suggesting that it may be jointly regulated by genes related to proline synthesis and metabolism.

**Figure 8:**
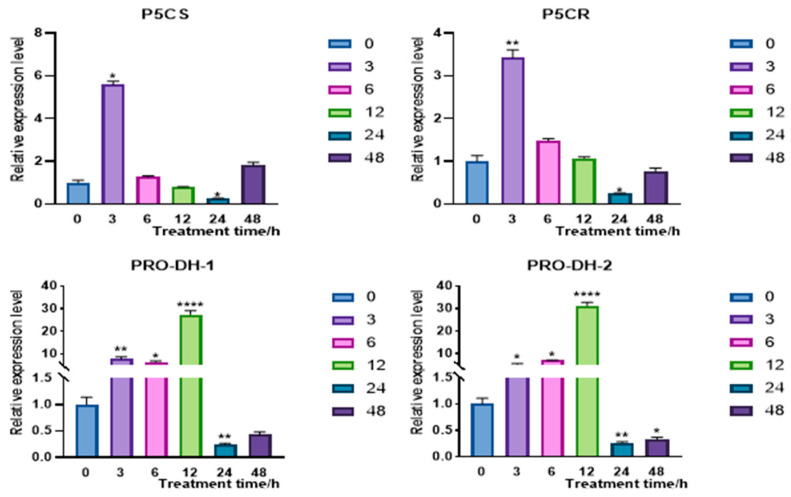
Expression patterns of proline metabolism-related genes in response to 15% PEG 6000 treatment. Actin1 was selected as the reference gene. *n* = 3. The asterisk (*) symbols indicate significant differences relative to 0 h, * *p* < 0.05, ** *p* < 0.01, **** *p* < 0.0001.

Therefore, dynamic expressions of key genes in the proline synthesis and catabolism pathway, such as *CqP5CS*, *CqP5CR*, *CqProDH1*, and *CqProDH2* under 15% PEG 6000 treatment, were analyzed utilizing qRT-PCR technologies. The expressions of *CqP5CS* and *CqP5CR* genes related to proline biosynthesis were 5.6- and 3.4-fold, respectively, reaching their peak at 3 h, indicating the proline synthesis pathway was rapidly activated in response to 15% PEG 6000 treatment, which is consistent with the early response mechanisms in response to abiotic stress [37]. Then, the expression of these two genes decreased to 25.5% and 24.7% at 24 h, respectively; after 48 h, the expressions returned to 1.8-fold and 76.3%, while the expression of *CqProDH1* and *CqProDH2* related to proline catabolism increased to 27.5 times and 31.2 times, respectively, reaching their peak at 12 h. They were down-regulated to 25.1% and 26.7%, respectively, at 24 h and rebounded to 42.8% and 32.6%, respectively, by 48 h (Figure 8). These results indicated that the expressions of *CqP5CS*, *CqP5CR*, *CqProDH1*, and *CqProDH2* exhibited a dynamic trend of initially increasing and then decreasing, which is negatively correlated with the expression of *CqPRX9L1*, with differences in response time and extent [38]. The peak of *CqProDH1* and *CqProDH2* occurred approximately 9 h later than that of *CqP5CS* and *CqP5CR*, but their increase was more significant. The expression analysis of key genes in the proline metabolism pathway showed that although proline catabolism genes (such as *CqProDH1* and *CqProDH2*) and synthesis genes (such as *CqP5CS* and *CqP5CR*) showed an up-regulation trend under 15% PEG 6000 treatment, the up-regulation of the catabolism pathway was significantly higher than that of the synthesis pathway, indicating that the proline catabolism pathway plays a more dominant regulatory role in the later stage of stress. These findings demonstrate that *CqPRX9L1* may dynamically regulate Pro content in response to abiotic stress through a dual regulatory mechanism of synergistically enhancing proline decomposition [39] and relatively inhibiting synthesis. These results revealed that *CqPRX9L1* may coordinate proline metabolism by regulating the time and intensity differences in synthesis and degradation pathways, which is consistent with previous research results [40]. This mechanism confirms the non-simple linear relationship between proline accumulation and plant stress resistance and provides a theoretical basis for understanding the complex regulatory network of plants in response to abiotic stress.

### 2.3. Subcellular Localization of CqPRX9L1 Protein

Under laser confocal microscopy, it was observed that the entire cell of *Arabidopsis thaliana* protoplasts transfected with green fluorescent protein empty vector control (pAN580: EGFP) exhibited green fluorescence signals, while the green fluorescence of GFP was distributed in the cytoplasm of protoplasts transfected with the target gene (pAN580: *CqPRX9L1*: EGFP), consistent with the predicted results on the website.

**Figure 9:**
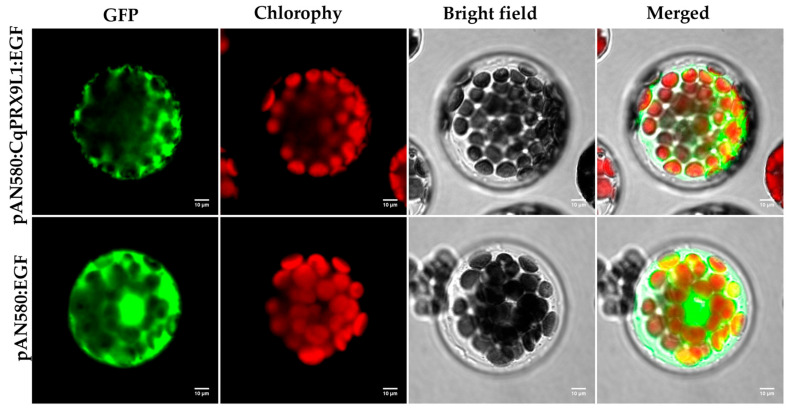
Subcellular localization of CqPRX9L1.The green fluorescence, red chloroplast spontaneous fluorescence, visible light, and merged images are presented.

Observation of *Arabidopsis* protoplasts under a laser confocal microscope revealed that the protoplasts containing the green fluorescent protein empty vector control (pAN580: EGFP) displayed green fluorescence signals throughout the cells, while the protoplasts containing the target gene pAN580: *CqPRX9L1*: EGFP showed GFP green fluorescence distribution in the cytoplasm (Figure 9), consistent with the predicted results on the website.

### 2.4. Expression Pattern of CqPRX9L1

#### 2.4.1. Overexpression of CqPRX9L1 Promotes Arabidopsis Plants to Bolt and Flower by Improving the Expression of Flowering Marker Genes

**Figure 10:**
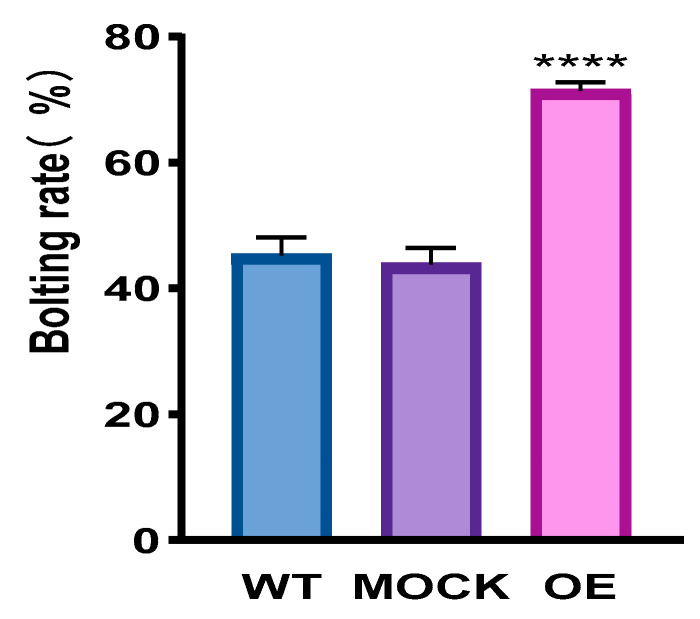
The bolting rate of wild-type and transgenic *Arabidopsis* plants. The asterisk (*) symbols indicate significant differences relative to WT. **** *p* < 0.0001. WT: wild type, MOCK: empty vector control, OE: *CqPRX9L1* transgenic lines.

**Figure 11:**
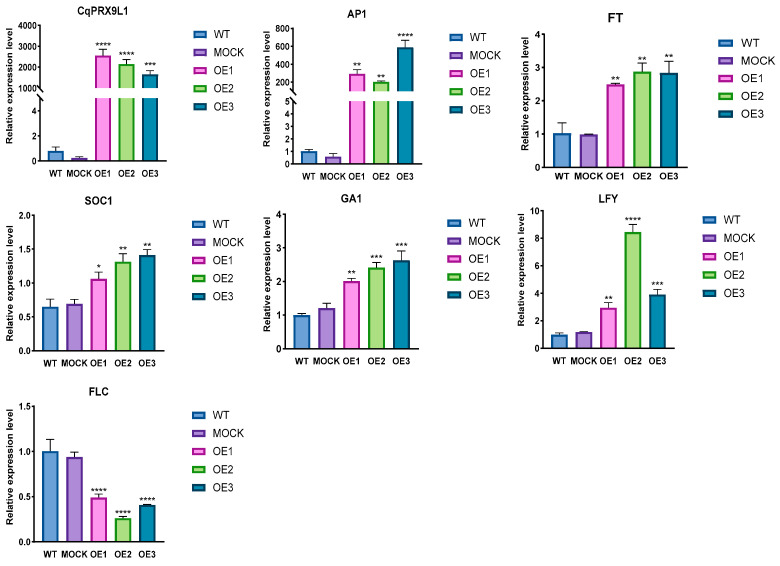
Differential expression of flower development-related genes in wild-type and transgenic *Arabidopsis* treated with 15% PEG 6000. Actin1 was selected as the reference gene. *n* = 3. The asterisk (*) symbols indicate significant differences relative to WT. * *p* < 0.05, ** *p* < 0.01, *** *p* < 0.001, **** *p* < 0.0001.

To further explore the role of *CqPRX9L1* in growth and development, overexpression lines in *Arabidopsis thaliana* were generated through homologous recombination of the PCR product of *CqPRX9L1* and the pCAMBIA1302 vector. The phenotype showed bolting and flowering of transgenics earlier than WT and MOCK under normal conditions. The bolting rates of WT, MOCK, and OE at the same growth time were statistically analyzed, and the results showed that the bolting rates of WT, MOCK, and OE were 45.3%, 43.8%, and 71.4%, respectively (Figure 10), indicating that overexpression of CqPRX9L1 promotes *Arabidopsis* bolting. In order to find out the reason and correlation for the earlier bolting and flowering of transgenics, expressions of flowering marker genes were analyzed, taking the 5th and 6th lotus leaves of *Arabidopsis thaliana* grown in the 5.10 period [41] as materials. The expression of *CqPRX9L1* was significantly higher than WT and MOCK, and the expressions of FT, GA, SOC1, LFY, and AP1 were significantly up-regulated compared to WT and MOCK, while the expression level of FLC was significantly down-regulated (Figure 11). Therefore, it is speculated that heterologous overexpression of *CqPRX9L1* in *Arabidopsis thaliana* may promote bolting and flowering by integrating photoperiod, autonomous pathways, and hormone signaling pathways to synergistically regulate the expression of flowering marker genes.

As demonstrated by Maria et al. (2019) [29], heterologous overexpression of *OsPRX38* in *Arabidopsis thaliana* significantly enhances superoxide dismutase (SOD) activity, suggesting a functional interplay between peroxidases (PRXs) and SODs in reactive oxygen species (ROS) scavenging. This PRX-SOD synergy likely establishes an optimal redox environment for developmental transitions, particularly flowering. The role of SODs in flowering regulation is further supported by Guo et al. (2023) [42], who showed that *TaCSOD* mainly regulates ROS homeostasis and flowering time through multiple interconnected pathways, including carbohydrate signaling, aging, vernalization, and gibberellin pathways.

#### 2.4.2. Overexpression of CqPRX9L1 Reduces Abiotic Stress Tolerance

**Figure 12:**
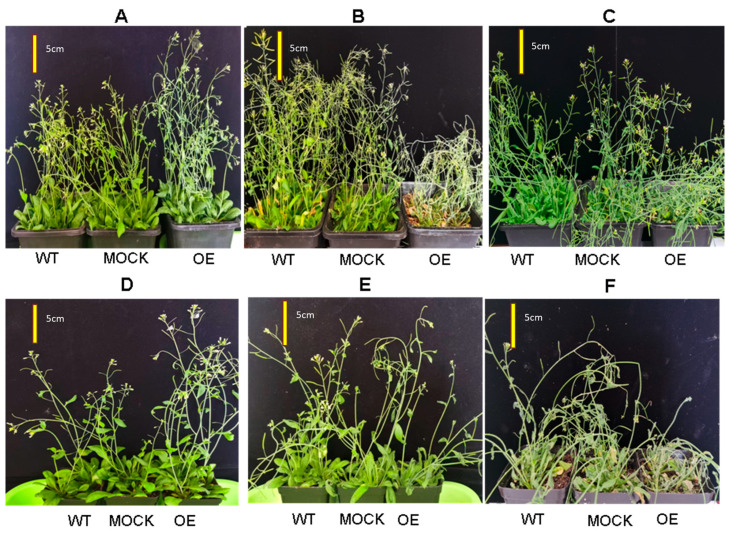
Phenotypic analysis of transgenic *Arabidopsis* lines after drought and rewatering treatment. WT: wild-type *Arabidopsis*. MOCK: transgenic *Arabidopsis* lines transformed with the empty vector (control). OE: *CqPRX9L1* transgenic lines. (**A**) Control. (**B**) No watering treated for 6 d. (**C**) Re-watering for 3 d. (**D**) Control. (**E**) 15% PEG 6000 treated for 4 d. (**F**) Re-watering for 4 d. Scale bar: 5 cm.

Under drought stress, transgenics are more sensitive than WT and MOCK; significant wilting of leaves and severe lodging of stems were observed after 6 days of no watering treatment, while WT and MOCK were less affected (Figure 12B) and then recovered after 3 days of rehydration (Figure 12C). When treated with 15% PEG 6000, transgenics are more sensitive than WT and MOCK. It is noteworthy that 15% PEG 6000-treated lines not only failed to recover but also exacerbated wilting.

To further explore the mechanism of *CqPRX9L1* in response to abiotic stress, the dynamic changes in the *CqPRX9L1* gene under 15% PEG 6000 treatment were analyzed, and stress resistance physiological indicators such as antioxidant enzyme activity, osmoregulatory substance content, and cell membrane stability were simultaneously detected.

**Figure 13:**
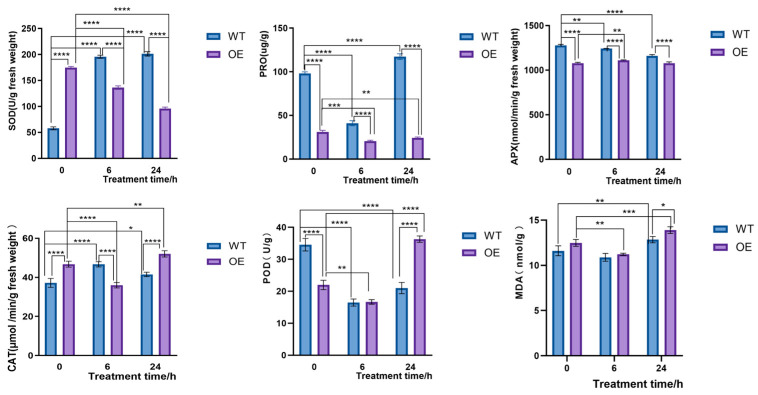

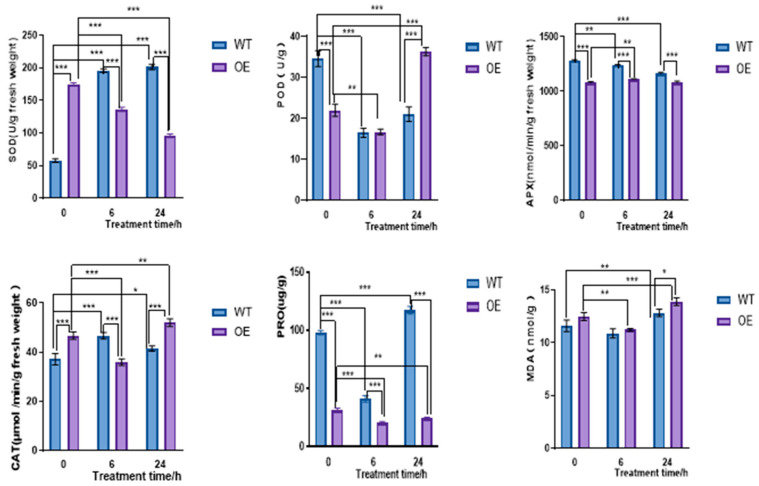
Physiological changes in transgenic *Arabidopsis* plants overexpressing *CqPRX9L1* from *Chenopodium quinoa* under 15% PEG 6000 treatment. *n* = 3. The asterisk (*) symbols indicate significant differences, * *p* < 0.05, ** *p* < 0.01, *** *p* < 0.001, **** *p* < 0.0001.

Under normal growth conditions, the POD and APX activities, as well as the proline (Pro) content, were significantly decreased to 63.6%, 84.3%, and 31.7% of WT in the overexpression lines, whereas the activities of SOD (superoxide dismutase) and CAT (catalase) were markedly increased (3.0-fold and 1.3-fold, respectively). Additionally, while the MDA content showed an increase (1.1-fold), it remained relatively stable during 24 h of 15% PEG 6000 treatment (Figure 13).

Notably, the dynamic changes in POD, SOD, CAT, and APX activities were closely associated with the expression pattern of *CqPRX9L1*, which aligns with the conclusion that *CqPRX9L1* acts as a negative regulator in stress responses, further supporting its critical regulatory role in stress adaptation.

**Figure 14:**
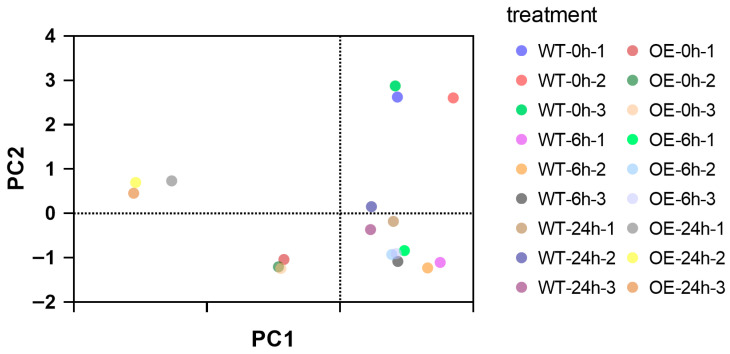
PCA of WT and OE lines under 15% PEG 6000 treatment at different time points. The dashed lines in the figure represent the 95% confidence intervals of each group of samples in principal component analysis (PCA).

Principal component analysis (PCA) results demonstrated tight clustering of biological replicates within each group, confirming high reproducibility. Under normal growth conditions (0 h stress), wild-type (WT) and *CqPRX9L1*-overexpressing (OE) lines exhibited clear separation, suggesting that differential expression of *CqPRX9L1* significantly alters baseline physiological states in plants. After 6 h of stress, WT samples showed pronounced divergence from their 0 h positions, indicating rapid stress-responsive physiological adjustments. In contrast, OE lines displayed minimal separation between 0 h and 6 h (Figure 14), implying that *CqPRX9L1* overexpression may stabilize early stress-induced physiological fluctuations. This buffering effect suggests that *CqPRX9L1* modulates stress signaling or regulatory pathways, potentially dampening initial stress perception and altering plant response dynamics. It showed that the sample points of the same group were tightly clustered, indicating that the reproducibility within the group was good. Under normal growth conditions (0 h of stress treatment), the samples of wild-type (WT) and overexpressed lines (OE) were significantly segregated, indicating that the difference in the expression of the *CqPRX9L1* gene would lead to significant differences in the physiological indexes of the two lines, suggesting that the gene may be involved in regulating the basic physiological state of plants. After 6 h of stress, the sample points of wild-type plants were significantly separated compared with those at 0 h, indicating that they could quickly respond to stress and induce physiological changes. However, the separation of OE lines at 0 h and 6 h was not obvious.

These findings demonstrate that these enzymatic adjustments failed to enhance stress tolerance and imply that *CqPRX9L1* functions as a negative regulator of stress adaptation, leading to a diminished ability to activate key defense mechanisms under adverse conditions, which are consistent with Liu et al.’s (2021) [21] hypothesis that overexpression of *OsPrx30* enhanced plant susceptibility to rice bacterial blight by maintaining high levels of POD activity and reducing H_2_O_2_. It showed the opposite effect when the expression of *OsPrx30* was suppressed, contrary to the study of Llorente et al. [26], where it was found that overexpressing *AtPrx3* showed an increase in dehydration and salt tolerance, whereas the antisense suppression of *AtPrx30* expression gave dehydration-sensitive and salt-sensitive phenotypes.

## 3. Materials and Methods

### 3.1. Test Materials

Quinoa Material: The quinoa cultivar used in this study is Huaqing44, provided by Shanxi Huaqing Quinoa Product Development Co., Ltd. (Taiyuan, China). The experiment was conducted using potted plants in the Dongyang Experimental Base of Shanxi Agricultural University (Shanxi Academy of Agricultural Sciences) (37°33′21″ N, 112°40′2″ E, 802 m above sea level). The organic matter content of the planting substrate is ≥35%, the pH is neutral, and it is mixed and divided into pots. The planting substrate, provided by Shenxian Luyuan Seedling Substrate Co., Ltd. (Liaocheng, China), had an organic matter content of ≥35% and a neutral pH. The substrate was thoroughly mixed and distributed into pots.

Arabidopsis material: Wild-type *Arabidopsis thaliana* seeds (WT, Columbia-0 ecotype, and Col-0) were stored in the laboratory. After sterilization, the seeds were dispersed and sown in 1/2 MS medium (Hope Bio-Technology Co., Ltd., Qingdao, China) [43] and placed in a 4 °C refrigerator for 48 h after being thoroughly rinsed with sterile water for 30 s, repeated disinfection 3 times, and, finally, rinsed with sterile water 3 times. The treated seeds were placed in an artificial climate chamber (YDRD-300L, Youke Instrument and Equipment Co., Ltd, Hefei, China). The light–dark period was 16 h of light and 8 h of darkness; the light intensity was 10,000 lx; the temperature was 22 °C; and the relative humidity was kept at 40%–60%. When the seedlings had grown 4 true leaves, they were transplanted to the substrate for further cultivation, covered with plastic wrap for 1 day, watered in time, and used for genetic transformation and phenotypic analysis in the later stage.

### 3.2. Bioinformatics Analysis of CqPRX9L1

The gene sequence, amino acid sequence, and homologous protein sequence with high similarity of *CqPRX9L1* (LOC_110724764) were obtained from the NCBI database (https://www.ncbi.nlm.nih.gov/) and the online cis-acting component analysis tool Plant CARE (http://bioinformatics.psb.ugent.be/webtools/plantcare/html/)was used to analyze the 2.5 kb sequences upstream of ATG and predict cis-acting elements. The Prot Param tool of the online analysis software ExPasy (http://web.expasy.org/protparam/) was utilized to analyze the number of amino acids encoded by the *CqPRX9L1* gene, the relative molecular weight, isoelectric point, and other physicochemical properties of the protein to predict its hydrophilicity and hydrophobicity. Transmembrane analysis on amino acid sequences was performed using DeepTMHMM (https://dtu.biolib.com/DeepTMHMM), and the conserved domain of CqPRX9L1 was analyzed using NCBI-CDD (https://www.ncbi.nlm.nih.gov/Structure/cdd/wrpsb.cgi). The secondary structure of CqPRX9L1 was predicted from SOPMA (https://npsa-prabi.ibcp.fr/cgi-bin/npsa_automat.pl?page=/NPSA/npsa_sopma_f.html), and subcellular localization of CqPRX9L1 was predicted using Cell Ploc (http://www.csbio.sjtu.edu.cn/bioinf/plant-multi/). Multiple sequence alignments were conducted using Clustal W in MEGA11 software, and phylogenetic trees were constructed with the same software employing the neighbor-joining (NJ) method [44], and bootstrap was set to 1000, and the rest of the parameters were kept as default.

### 3.3. Subcellular Localization of CqPRX9L1

#### 3.3.1. Construction of Expression Vectors

**Figure 15:**
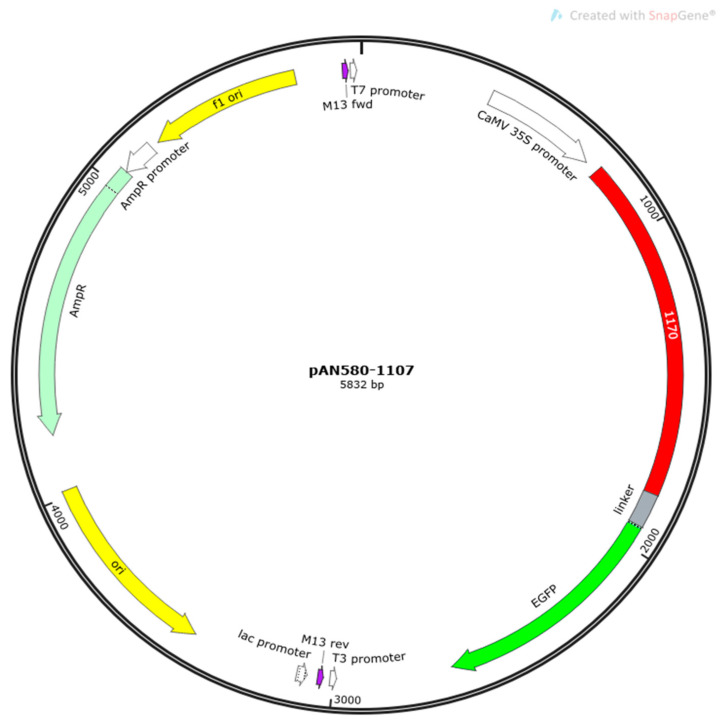
Construction of subcellular localization vector for CqPRX9L1-GFP fusion. The arrow represents the transcription direction of the gene, and the red one indicates the target gene, dark green is the eGFP reporter gene, and light green represents a common screening marker AmpR in vectors.

The *CqPRX9L1* coding sequence was amplified from quinoa cDNA using BioRun Pfu PCR Mix (BioRun, Wuhan, China) with gene-specific primers containing 15–20 bp extensions homologous to the linearized pAN580 vector (Figure 15).

Homologous recombination was performed using the BioRun Seamless Cloning Kit (BioRun, Wuhan, China). Briefly, 50 ng of linearized vector was mixed with 100 ng of purified PCR product in 2× EasyClone mix and incubated at 37 °C for 30 h to allow homologous end joining. The reaction mixture was then transformed into *Escherichia coli* (*E. coli*) DH5α competent cells by electroporation [45]. Transformed cells were plated on LB agar containing 50 μg/mL ampicillin and incubated at 37 °C for 12 h. Positive clones were selected and verified by colony PCR and Sanger sequencing. The specific primers used for gene cloning are listed in Table 3.

#### 3.3.2. Transformation of Arabidopsis Protoplasts

*Arabidopsis* protoplasts were extracted and transformed [46]. Transformed protoplasts were cultured in the dark at 28 °C for 18–24 h, and GFP fluorescence was observed using a Nikon C2 laser scanning confocal microscope (Nikon Corporation, Tokyo, Japan). GFP signals were captured using a 10× eyepiece and 100× oil immersion objective, with excitation at 488 nm and emission collected at 510 nm, while chlorophyll autofluorescence was detected with excitation at 640 nm and emission at 675 nm. Images were acquired using Image J-win 64 (NIH, Bethesda, MD, USA) with identical exposure settings for all samples, and the fluorescence patterns were analyzed to determine the subcellular localization of the target protein, with GFP fluorescence indicating successful expression and proper folding of the fusion protein.

### 3.4. Expression Pattern of CqPRX9L1 in Quinoa

#### 3.4.1. Spatiotemporal Expression Pattern of CqPRX9L1

Total RNA was isolated from roots, stems, leaves, and spikes (1.3–1.8 cm) of quinoa during the tasseling stage using the RNAprep Pure Plant RNA Purification Kit (Tiangen, Beijing, China). The concentration and purity of DNA/RNA samples were measured by a micro nucleic acid analyzer (Tnano-700, Tuohe Electromechanical Technology Co., Ltd., Shanghai, China) with A260/A280 ratios between 1.8 and 2.0 and A260/A230 ratios exceeding 2.0, while visualization on 1.2% agarose gel electrophoresis demonstrated intact 28S and 18S ribosomal RNA bands. Furthermore, RNA integrity was quantitatively verified using the Bioanalyzer 2100 system (Agilent Technologies, Santa Clara, CA, USA), with all samples exhibiting RNA integrity numbers (RIN) greater than 7.0, indicating high-quality RNA suitable for downstream applications.

First-strand cDNA was synthesized using the HiScript^®^ll Q RT SuperMix for qPCR (+gDNA wiper) (TransGen Biotech, Beijing, China). Quantitative reverse-transcription polymerase chain reaction (qRT-PCR) analysis was conducted using the ChamQ Universal SYBR qPCR Master Mix (Vazyme, Nanjing, China) to prepare the reaction mixture, which was analyzed with the Roche Light Cycler480 real-time fluorescence quantitative PCR instrument to detect the spatiotemporal expression pattern of *CqPRX9L1.* Gene expression levels were analyzed using the 2^−ΔΔCt^ method [47]. The length of the *CqPRX9L1* PCR product was 113 bp, and the reference gene actin1 (165 bp) was used in qRT-PCR studies, providing reliable normalization for gene expression analysis in this experiment.

#### 3.4.2. Expression Patterns and Functional Analysis of CqPRX9L1 Under Different Stress Treatments

Potted quinoa plants at the spiking stage were treated with 15% PEG 6000, low temperature at 4 °C, and 100 mM NaCl, and total RNA was extracted from leaves. The expression of *CqPRX9L1* under different stresses was detected by qRT-PCR. At the same time, synchronous sampling was conducted on quinoa leaves treated with 15% PEG 6000, and drought-related physiological indicators such as SOD activity, POD activity, proline content, and MDA content, and the correlation between gene expression and physiological response mechanisms under drought stress were analyzed. The detection methods for the above-mentioned indicators are described in Section 3.7.

### 3.5. Construction of Plant Heterologous Overexpression Vector CAM-CqPRX9L1-GFP and Genetic Transformation of Arabidopsis thaliana

The *CqPRX9L1* coding sequence was PCR-amplified and cloned into the CAM-GFP control (CAM-GFP), and the fusion construct was transformed into Agrobacterium tumefaciens strain GV3101 for plant transformation. *Arabidopsis thaliana* (Col-0) inflorescences were transformed using the floral dip method [48]. Agrobacterium tumefaciens containing CAM-GFP and CAM-*CqPRX9L1*-GFP plasmids were cultivated, using the floc staining method to stain *Arabidopsis* inflorescences. T_0_ seeds were collected, and T_1_ transformants were selected on 1/2 MS medium supplemented with 50 mg/L streptomycin. Homozygous T_3_ lines were subsequently obtained through two additional generations of antibiotic selection and molecular verification.

### 3.6. Physiological Analysis of Drought Stress Responses

Physiological parameters associated with drought resistance were quantified in quinoa plants and *CqPRX9L1*-overexpressing transgenic *Arabidopsis* lines subjected to 15% PEG 6000 treatment. All assays were performed using Wilmin biochemical detection kits (Wilmin Biotechnology Co., Ltd., Changzhou, China) according to the manufacturer’s protocols. We performed all measurements using 96-well ELISA plates (Corning Inc., Corning, NY, USA) with strict quality control measures to ensure data reliability. Before sample analysis, we conducted plate calibration and verified standard curve linearity (R^2^ > 0.99), while maintaining inter-well optical density variation below 0.002 at the assay wavelength. Each run included appropriate blank controls, standard references, and internal quality control samples. Absorbance readings were taken at assay-specific wavelengths with three technical replicates per biological sample using a Multiskan GO microplate reader (Thermo Fisher Scientific, Waltham, MA, USA), and data were analyzed with SkanIt Software 6.0.

#### 3.6.1. Peroxidase (POD) Activity Assay

(1) Sample pretreatment: Fresh leaf tissue (0.1 g) was homogenized in 1 mL ice-cold extraction buffer using a pre-chilled mortar and pestle. The homogenate was centrifuged at 12,000 rpm for 10 min at 4 °C, and the resulting supernatant was immediately placed on ice for subsequent analysis.
(2) In this study, 10 µL of supernatant and 190 µL of working solution were mixed well, and the absorbance A1 at 470 nm for 10 s and the absorbance A2 at 470 nm for 1 min and 10 s were recorded; ΔA = A2 − A1 is calculated; If the absorbance value at 1 min and 10 s is greater than 1.5 or ΔA is greater than 0.5, the sample can be diluted with extraction solution for detection; If ΔA is less than 0.005, the reaction time can be extended to 5 min.
(3) Quantitative unit definition:

One unit (U) of enzyme activity is defined as the amount of enzyme required to produce a change in absorbance at 470 nm of 0.5 per minute in a 1 mL reaction system containing 1 g of fresh tissue.

$$\mathrm{POD}\;(U/g\;\mathrm{fresh weight})\;=\;\frac{\Delta A\times \mathrm{Vt}\times T╳100\%}{\mathrm{Vs}\times W\times 0.5}\;=\;\frac{40╳\Delta A}{W}$$

where Vt is the total volume of the reaction system, 0.2 mL; Vs is the added sample volume, 0.01 mL; T is the reaction time, 1 min; and W is the sample quality, g.

#### 3.6.2. Superoxide Dismutase (SOD) Activity Assay

(1) Sample pretreatment: Fresh leaf tissue (0.1 g) was homogenized in 1 mL ice-cold extraction buffer using a pre-chilled mortar and pestle. The homogenate was centrifuged at 12,000 rpm for 10 min at 4° C, and the resulting supernatant was immediately placed on ice for subsequent analysis.
(2) In this study, 10 µL of supernatant (distilled water was taken as a control), 10 µL of reagent 3, 20 µL of reagent 4, and 160 µL of working solution were mixed well and incubated at room temperature (25 °C) for 30 min, and the absorbance value A of each tube at 450 nm was recorded. Cautionary notes: Reagent 3 contains thermolabile enzymes and must not be frozen. Keep on ice during use.
(3) Quantitative results

$$\mathrm{Inhibition percentage}=\;\frac{\mathrm{Ac}-\mathrm{At}╳100\%}{Ac}$$

Try to keep the inhibition percentage of the sample within the range of 20–80%. If the calculated inhibition percentage is less than 20% or greater than 80%, it is usually necessary to adjust the sample size and retest. If the measured inhibition percentage is too high, the sample needs to be appropriately diluted with an extraction solution. If the measured inhibition percentage is low, it is necessary to prepare a sample with a higher concentration for testing again.

$$\mathrm{SOD activity}\;(U/g\;\mathrm{fresh weight})\;=\;\frac{\mathrm{Inhibition}\;\mathrm{percentage}\times V\;\mathrm{tr}\times \mathrm{Vts}}{\left(1-\mathrm{Inhibition}\;\mathrm{percentage}\right)\times W\times \mathrm{Vs}}\;=\;\frac{20\times \mathrm{Inhibition}\;\mathrm{percentage}}{\left(1-\mathrm{Inhibition}\;\mathrm{percentage}\right)}$$

where Vtr is the total volume of the reaction system, 0.2 mL; Vts is the extraction solution volume, 1 mL; Vs is the sample volume added to the reaction system, 0.01 mL; and W is the sample quality, g.

#### 3.6.3. Ascorbate Peroxidase (APX) Activity Assay

(1) Sample pretreatment: Fresh leaf tissue (0.5 g) was homogenized in 1 mL ice-cold extraction buffer using a pre-chilled mortar and pestle. The homogenate was centrifuged at 12,000 rpm for 10 min at 4 °C, and the resulting supernatant was immediately placed on ice for subsequent analysis.
(2) In this study, 10 µL of supernatant, 140 µL of reagent 1, 20 µL of reagent 2, and 30 µL of diluted reagent 3 were mixed well, and the absorbance values A1 and A2 at 290 nm for 10 s and 130 s were recorded; ΔA = A2 − A1 is calculated. If a large number of bubbles are generated during the reaction process, it indicates high enzyme activity. The supernatant can be diluted before measurement. If ΔA is negative and the value is small, the reaction time can be extended.
(3) Quantitative definition of active unit:

An aliquot of 1 nmol of AsA is oxidized per minute per g of tissue, which is equivalent to one enzyme activity unit.

$$\mathrm{APX}(\mathrm{nmol}/\min/\mathrm{mg}\;\mathrm{prot})\;=\;\frac{\left(△A\times \mathrm{Vtr}\times 10^{9}\times \mathrm{Vts}\right)}{(\varepsilon \times d)\times \left(W\times \mathrm{Vs}\right)\times T}\;=\;\frac{3571\times △A}{W}$$

where:

ε is the molar absorptivity of AsA at 290 nm, 2.8 × 10^3^ L/mol/cm;
d is the 96-well plate optical diameter (cm), 0.5 cm;
Vtr is the total volume of the reaction system (L);
200 μL = 2 × 10^−4^ L;
10^9^: 1 mol = 1 × 10^9^ nmol;
W is the sample quality;
Vs is the volume of supernatant (mL) added to the reaction system;
20 μL = 0.02 mL;
Vts is the extraction volume, 1 mL;
T is the catalytic reaction time (min), 2 min.

#### 3.6.4. Catalase (CAT) Activity Assay

(1) Sample pretreatment: Fresh leaf tissue (0.5 g) was homogenized in 1 mL ice-cold extraction buffer using a pre-chilled mortar and pestle. The homogenate was centrifuged at 12,000 rpm for 10 min at 4 °C, and the resulting supernatant was immediately placed on ice for subsequent analysis.
(2) In this study, 10 µL of supernatant and 190 µL of the working solution were mixed well, and the absorbance values A1 at 240 nm for 10 s and A2 for 1 min and 10 s were recorded; and ΔA = A2 − A1 is calculated; Note: If ΔA is negative during detection, a kinetic cycle can be set (To measure absorbance every 30 s or 1 min for 5–10 min), and the absorbance difference within a 1-minute interval in the decreasing range should be taken. If visible bubbles are observed after the reaction, it indicates high sample activity, and the sample can be diluted before detection. For a large number of samples, a multichannel pipette can be used to add the working solution.
(3) Quantitative results

$$\mathrm{CAT}(\mu \mathrm{mol}/\min/\mathrm{mL})\;=\frac{\Delta A\times \mathrm{Vtr}\times 10^{6}\times T\times \mathrm{Vts}}{(\varepsilon \times d)\times W\times \mathrm{Vs}}$$

where:

Vtr is the total volume of the reaction system, 2 × 10^−4^ L;
Ε is the molar extinction coefficient of H_2_O_2_, 43.6 L/mol/cm;
D is the 96-well plate optical diameter, 0.5 cm;
Vs is the added sample volume, 0.01 mL;
Vts is the added extraction solution volume, 1 mL;
T is the reaction time, 1 min;
W is the sample quality, g.

#### 3.6.5. Determination of Proline Content

(1) Sample pretreatment: Fresh leaf tissue (0.05 g) was homogenized in 0.5 mL extraction buffer in a 90 °C water bath for 10 min. The homogenate was centrifuged at 10,000 rpm for 10 min at 25 °C, and the resulting supernatant was immediately placed on ice for subsequent analysis.
(2) In this study, 250 µL of supernatant (or 250 µL of extract as control), 250 µL of reagent 1, and 250 µL of reagent 2 were mixed well and placed in a 92 °C water bath for 30 min (covered tightly to prevent water loss), cooled down, 500 µL of reagent 3 was added, and shaken for 30 s, and then the pigment was transferred to reagent 3. Then, 200 μL of the upper solution was sucked into a 96-well plate, A520 was measured, and ΔA = Am − Ab was calculated. If ΔA is greater than 1, the supernatant was diluted with the extraction solution for measurement and multiplied by the dilution factor when calculating. If ΔA is less than 0.0047, it is recommended to weigh 0.2 g of sample and add 1 mL of extraction solution for concentration treatment.
(3) Quantitative results

$$\mathrm{Proline content}(\mu \;g/g\;\mathrm{fresh weight})\;=\;\frac{(△A-0.0047)╳V1}{0.0209╳W\;╳V1}╳V2\;=\;\frac{(△A-0.0047)╳47.85}{W\;}╳0.5$$

where:

V1 is the sample volume added to the reaction system, 0.25 mL;
V2 is the extract volume added, 0.5 mL;
W is the sample quality, g;
Am is the A measure;
Ab is the A blank.

#### 3.6.6. Determination of MDA Content

(1) Sample pretreatment: Fresh leaf tissue (0.1 g) was homogenized in 1 mL extraction buffer, the mixture was homogenized at room temperature and centrifuged at a speed of 10,000 rpm for 10 min at room temperature, and the supernatant was taken for testing.
(2) In this study, 100 µL of supernatant and 300 µL of reagent 2 were mixed well and water-bathed at 92 °C for 30 min (covered tightly to prevent water loss); after cooling, they were centrifuged at a speed of 12,000 rpm at 25 °C for 10 min. Then, 200 μL of supernatant was taken, and the absorbance values A at 532 nm and 600 nm were measured in a 96-well plate, denoted as A532 and A600, with ΔA = A532 − A600. If ΔA ≤ 0, it indicates that the MDA content of the sample is low, and the sampling quality can be increased, such as 0.2 g plus 1 mL of extraction solution.
(3) Quantitative results

$$\mathrm{MDA content}\;(\mathrm{nmol}/g)\;=\;\frac{\left(△A+0.0055\right)\times V}{0.025\times W}\;=\;\frac{\left(△A+0.0055\right)\times 40}{W}$$

where V is the volume of extraction solution and 1 mL W is the sample mass, g.

#### 3.6.7. Determination of H_2_O_2_ Content

(1) Sample pretreatment: Fresh leaf tissue (0.1 g) was homogenized in 1 mL TCA, the mixture was homogenized at 4 °C, and centrifuged at a speed of 12,000 rpm for 15 min.
(2) In this study, 200 µL of supernatant, 200 µL of phosphate buffer, and 400 µL of KI were mixed, vortexed thoroughly, and incubated for 10 min, and the OD value at 390 nm was measured.
(3) Quantitative results

$$H_{2}O_{2}\;\mathrm{content}\;(\mathrm{nmol}/g)\;=\;\frac{C\times V}{M}$$

where C is the result calculated based on the standard curve; M is the actual measured mass; and V is the total volume of the extraction solution.

### 3.7. Statistical Analyses

Statistical comparisons were performed using GraphPad Prism 10.4.2 [49]. The data were analyzed by a two-tailed Student’s *t*-test or one-way ANOVA followed by Duncan’s multiple comparisons test, with statistical significance set at *p* < 0.05. Significant differences relative to the control (0 h) are indicated as follows: * *p* < 0.05, ** *p* < 0.01, *** *p* < 0.001, and **** *p* < 0.0001.

## 4. Conclusions

In this study, a comprehensive study of the Cq*PRX9L1* gene in quinoa was carried out. The results of bioinformatics analysis showed that the promoter region of the Cq*PRX9L1* gene contained light-responsive elements; stress-response elements, including MBS, LTR, DRE1, W box, etc.; and hormone-response elements, including TCA element, ABRE, TGACG motif, and other hormone-response elements. The meristem-specific element CAT box suggests that the *CqPRX9L1* gene may be regulated by light induction and plant hormones and is related to tissue atopic expression and stress response (Table 2). Amino acid sequence alignment and phylogenetic tree analysis of CqPRX9L1 and other homologous proteins showed that CqPRX9L1 was most closely related to spinach (*Spinacia oleracea*) (So PRX9, XP_021851032.1) and sugar beet (*Beta vulgaris*) (Bv PRX9, XP_010672794.2).

*CqPRX9L1* exhibits distinct tissue-specific expression patterns, showing significantly higher transcript levels in spikes (25.7-fold increase) and root tissues (14.3-fold increase) compared to other tissues. This pronounced differential expression suggests its potential functional roles in both inflorescence development and root-specific physiological processes.

*CqPRX9L1* also affects the flowering process of plants by regulating key flowering genes (such as FT and AP1) and gibberellin (GA)-related pathways. *CqPRX9L1* showed a dynamic expression pattern of first decreasing and then increasing under abiotic stresses such as 15% PEG 6000, low temperature, and salt damage, with differences in response time and degree. CqPRX9L1 can play a role in the process of stress resistance by affecting the activity of antioxidant enzymes such as superoxide dismutase (SOD) and peroxidase (POD), as well as the synthesis and decomposition of proline (Pro). 

## Figures and Tables

**Table 1:** Statistical results of differentially expressed genes (DEGs).

No.	Compare Group	Up-Regulated Genes	Down-Regulated Genes	Total
1	d5 vs. ck	708	583	1291
2	d10 vs. ck	1068	821	1889
3	d15 vs. ck	1971	3382	5353
4	D20 vs. ck	3313	5678	8991

**Table 2:** Analysis of promoter components.

Cis-Element	Location	Sequence	Function
I box	−1377 to −1366	CCATATTCAATA	Photoinduction element
W box	−602 to −597	TTGACC	Participate in multiple stress responses collaborating with WRKY
ERE	−948 to −941	ATTTTAAA	Ethylene-responsive element
DRE1	−541 to −535	ACCGAGA	Dehydration-response element
ARE	−401 to −396	AAACCA	Anaerobic induction element
MBS	−2322 to −2317	CAACTG	MYB binding sites related to drought induction
TCT motif	−2395 to −2390, −732 to −727	TCTTAC	Photoreactive element
TCA element	−1604 to −1595	TCAAAAGAGG	Elements related to salicylic acid reaction
TGACG motif	−1589 to −1585	TGACG	MeJA
ATCT motif	−395 to −387	AACTAATCC	Conservative structural domains related to photoreaction
CAT box	−1822 to −1817	GCCACT	Elements related to meristem expression
MRE	−1512 to −1506	AACCTAA	MYB binding sites related to Photoreaction
box4	−1420 to −1415	ATTAAT	Conservative structural domains related to photoreaction
ABRE	−579 to −575	ACGTG	Abscisic acid reaction element
LTR	−558 to −552	CCGAAA	Low-temperature reaction element
ATC motif	−357 to −350	AGTAATCT	Conservative structural domains related to photoreaction

**Table 3:** Primers used in this study.

Primer Names	Primer Sequences (5′→3′)
pAN580 F	AAGTCCGGAGCTAGCTCTAGATGGCTACAAAAATGCACCTAGTGTTTTTGGC
pAN580 R	AGCGGCCGCTGTACAGGATCATTAGGGCGACGACAATACTTCCTAACTTC
CAM-FLAG-GFP F	TGGAGAGGACACGCTCGAGATGGCTACAAAAATGCACCTAGT
CAM-FLAG-GFP R	CATCCTTGTAGTCGAATTCATTAGGGCGACGACAATACTTC
CAM-FLAG-GFP-PF	AGAAGACGTTCCAACCACG
CAM-FLAG-GFP-PR	CGGTAAGGATCTGAGCTACAC
CAM-FLAG-GFP-TF	TGACGCACAATCCCACTATC
CAM-FLAG-GFP-TR	GCGACAAGATCAACTTCATCTAG
CqproDH-1-F	CCGACAAGAAGAACAAGACCTT
CqproDH-1-R	CGCCGAGTTATACGTCAAGTAA
CqproDH-2-F	CAGTCCTTGCCACCCATAATG
CqproDH-2-R	CGCCTCCGACATTCCGTAT
Cqp5CR-F	GGATGTGGTGTTACAGTTGAGA
Cqp5CR-R	GCAGCAGGAGTATTAGGCATTA
CqP5CS-F	GGAGAATGGCACTTGGAAGAC
CqP5CS-R	TGACGACCAGCACTGACAG
CqACT1-F	GTCCACAGAAAGTGCTTCTAAG
CqACT1-R	AACAACTCCTCACCTTCTCAT

## Data Availability

The data presented in this study are available on request from the corresponding author.
